# A Tablet-Based Mobile Hearing Screening System for Preschoolers: Design and Validation Study

**DOI:** 10.2196/mhealth.9560

**Published:** 2018-10-23

**Authors:** Kwanchanok Yimtae, Pasin Israsena, Panida Thanawirattananit, Sangvorn Seesutas, Siwat Saibua, Pornthep Kasemsiri, Anukool Noymai, Tharapong Soonrach

**Affiliations:** 1 Department of Otorhinolaryngology Faculty of Medicine Khon Kaen University Khon Kaen Thailand; 2 National Electronics and Computer Technology Center National Science and Technology Development Agency Klong Luang, Pathumthani Thailand

**Keywords:** hearing screening, mobile health, speech audiometry, hearing loss

## Abstract

**Background:**

Hearing ability is important for children to develop speech and language skills as they grow. After a mandatory newborn hearing screening, group or mass screening of children at later ages, such as at preschool age, is often practiced. For this practice to be effective and accessible in low-resource countries such as Thailand, innovative enabling tools that make use of pervasive mobile and smartphone technology should be considered.

**Objective:**

This study aims to develop a cost-effective, tablet-based hearing screening system that can perform a rapid minimal speech recognition level test.

**Methods:**

An Android-based screening app was developed. The screening protocol involved asking children to choose pictures corresponding to a set of predefined words heard at various sound levels offered in a specifically designed sequence. For the app, the set of words was validated, and their corresponding speech power levels were calibrated. We recruited 122 children, aged 4-5 years, during the development phase. Another 63 children of the same age were screened for their hearing abilities using the app in version 2. The results in terms of the sensitivity and specificity were compared with those measured using the conventional audiometric equipment.

**Results:**

For screening purposes, the sensitivity of the developed screening system version 2 was 76.67% (95% CI 59.07-88.21), and the specificity was 95.83% (95% CI 89.77-98.37) for screening children with mild hearing loss (pure-tone average threshold at 1, 2, and 4 kHz, >20 dB). The time taken for the screening of each child was 150.52 (SD 19.07) seconds (95% CI 145.71-155.32 seconds). The average time used for conventional play audiometry was 11.79 (SD 3.66) minutes (95% CI 10.85-12.71 minutes).

**Conclusions:**

This study shows the potential use of a tablet-based system for rapid and mobile hearing screening. The system was shown to have good overall sensitivity and specificity. Overall, the idea can be easily adopted for systems based on other languages.

## Introduction

Hearing loss in one of the most common disabilities. The World Health Organization estimated that, in 2012, there were 360 million people in the world with disabling hearing loss (5.3% of the world’s population); 328 million (91%) were adults, and 32 million (9%) were children [1]. The incidence of permanent congenital hearing loss, or childhood hearing loss, in low- and middle-income countries can be 3 times higher than the incidence in high-income regions [2]. Among all types of hearing loss, the sensorineural hearing loss is the most common. In this case, the damaged hair cells of the inner ear diminish sounds to be effectively converted into nerve signals to the brain. Unfortunately, the sensorineural hearing loss cannot be reversed, and the patient is usually advised to use a hearing aid or a cochlear implant in the case of profound hearing loss. Other types of hearing loss include conductive and mixed hearing losses. Conductive loss occurs when there is an obstruction of, or damage to, the outer or middle ear that blocks sound from being conducted to the inner ear. The conductive hearing loss may be temporary or permanent depending on the cause, sometimes requiring medical or surgical treatments to improve the hearing ability of those affected. The final type of hearing loss is a mixed hearing loss, which is a combination of sensorineural and conductive hearing loss. Even though the universal hearing screening can detect permanent neonatal hearing loss, it does not identify late-onset, acquired, or many cases of progressive hearing loss. Approximately 60% of childhood hearing loss is due to preventable causes such as otitis media, recreational noise-induced hearing loss, and ototoxicity. Early detection and intervention are, therefore, extremely important in children, as hearing loss affects the ability to learn and hinders crucial language, speech, and emotional development [2]. Learning tonal languages, such as Thai in particular, can be greatly affected in children with hearing difficulties, as described previously [3]. Therefore, preschool and school hearing screening are effective tools for early identification and management of childhood hearing loss [4].

Hearing testing in children is challenging and usually requires specialist supervision. Depending on the age, each child can have varying capability to cooperate. For newborn up to 3 years of age, objective measurements, such as otoacoustic emission or auditory brainstem response, may be more appropriate [5]. The objective tests measure how the ear or nerve respond to sounds; these tests are sufficiently sensitive to diagnose hearing abnormalities but cannot represent the true hearing threshold. Subjective tests, such as pure-tone audiometry, which is the gold standard for measuring hearing acuity, are usually considered for older children who can provide appropriate responses [6]. The reliability of pure-tone audiometry depends on the machines, noise environments, experience of test operators, and pupils.

Many strategies have been attempted to make the preschool or school-age hearing screening feasible, fast, accurate, and cost-effective; however, to date, these strategies have not managed to meet the target goal [7-12]. The most widely preferred, and still considered the gold standard hearing screening in 4-6-year-old children, is the pure-tone audiometric sweep test [8-13]. In limited-resource countries, implementing this method nationwide can be a challenge. Furthermore, as the abstraction of pure-tone is difficult to understand, the response to pure tones in preschool or school-aged children may not as effective or reliable as speech [14]. For speech screening, the Verbal Auditory Screening for Children of Griffing et al has been used since 1962. However, the Verbal Auditory Screening for Children failed to identify children with mild hearing loss [15]. Another disadvantage of speech audiometry is that the results are not frequency-specific, especially for high-frequency hearing loss. However, the advantage of speech screening is that it assesses the auditory perceptual development, composed of sound awareness, phonetic discrimination, and word recognition [16]. Many techniques of speech audiometric tests have been developed; some were interfered with by nonauditory influences, such as maturity, experience, processing skills, and motor skills, of children [16]. Furthermore, young toddlers could be more responsive to a social interaction, particularly with their caregivers. Therefore, integrating auditory testing into a type of scripted interaction between child and caregiver could be more successful [16]. As such, even though they tend to be superior to pure-tone audiometry, such testing is not comparable with the standard one in large groups [17,18].

Recent advances in wireless telecommunication have made great changes in public health practice. A mobile app can potentially change a smartphone or a tablet into a medical device. These mobile devices are becoming increasingly powerful while the costs are becoming progressively lower, and the pervasive nature of the existing network answers the accessibility question. Hearing screening and measurement could be considered one of the early apps in the age of mobile or digital health care [19]. Some reports discuss this approach for individual hearing screening. Davison et al showed the effectiveness of using tablet-based hearing screening system compared with that of traditional audiometry for populations aged >60 years [20]. He proposed and validated the ability to use the tablet for a hearing assessment. Shouneez et al investigated the community-based identification of hearing loss using smartphones [21] and found that smartphone-based hearing screening allowed community health workers to bring hearing health care to underserved communities at the primary care level. Rourke et al used portable tablets to test hearing loss of 218 children in Northern Canadian communities [22]. The study provided positive and valuable evidence for using the tablet-based audiometer in remote areas. Whitton et al compared hearing measurements made at home using self-administered audiometric software against the standard tests in clinical settings [23]; the results showed statistical equivalence between the 2 approaches. Samelli et al confirmed the results of Whitton et al [24].

This study was designed to develop an appropriate tool to be used as a hearing screening device for preschool children. The system was implemented on an Android-based tablet, details of which are discussed in the following sections.

## Methods

### Overview

The study was approved by the Khon Kaen University Ethical Committee for Human Research (HE 571278) and was registered in the Thai Clinical trial (TCTR2014092201). This study was composed of 2 phases. The first phase was the development of the speech audiometry app software and was conducted during 2015-2016. The second phase was the validity of the software and was conducted during 2016-2017. The details of each phase are provided below.

### Development of the Speech Audiometry App Software

#### Concept Design

#### Word Selection

To select appropriate words for the screening device, first, an audiologist in our research team chose 36 two-syllable words from an elementary school book that varied in terms of pitch. Corresponding pictures of the words were drawn with the consultation of 3 external audiologists. These words and corresponding pictures were then tried out with 2 groups of 30 preschool children aged between 4 and 5 years. One group was recruited from an urban area, and another group was recruited from a rural area. Parental consents were obtained before the test date. In addition, verbal child assent was also obtained. Each child would first be presented with the set of 6 pictures. There were 6 sets of pictures; these pictures were grouped by category as things, actions, fruits, etc. The researcher pronounced only one word at a time in a random order. A child was asked to point to the picture that represented the word they heard. The number of times the child correctly identified the picture was noted. After completing the 6 sets, the child was again presented with the pictures, this time one by one. They were asked what the picture was and the answer was recorded. The 24 words that 95% of children recognized correctly would be used for the software.

#### Sound Recording

All recordings were made in a sound-proof booth at a music studio. A high-sensitivity microphone (Brüel & Kjær type 4188) was positioned approximately 20 cm from the speaker at 0° azimuth and was covered with a 6.5-cm diameter windscreen. The microphone was connected to a sound level meter (B & K model 2239A), and the signal from the linear-weighted AC output of this meter was fed to an analog-to-digital converter (National Instruments, model PXI-4461) that acquired the mono sounds at a sampling frequency of 44.1 kHz with 16-bit amplitude resolution. A female speaker, who was a professional announcer with 25 years’ experience, enunciated with normal vocal effort, corresponding to approximately 63 decibels sound pressure level (dBSPL; as monitored by the sound level meter) and recorded at a sampling rate of 44.1 kHz.

The sound intensity of each word was edited to yield the same average intensity as that of a 1000-Hz calibration tone.

#### System

**Figure 1:**
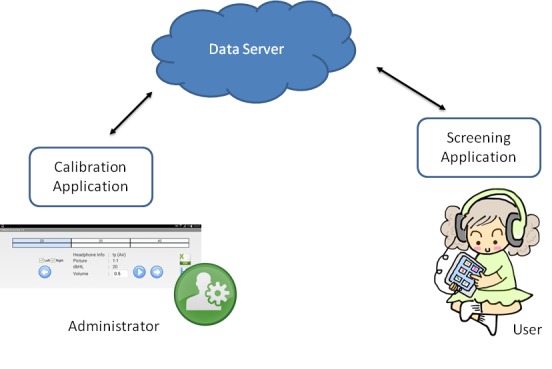
System overview.

The system consisted of a tablet device running 2 Android apps. We used the Samsung Galaxy Tab S 10.1. The tablet was plugged in with an approved headphone to be worn by the child being tested. The app “Calibrate Screening” was used by the support team to provide an Web-based update to the library of calibrated headphones that are selectable within the “Screening” app. Figure 1 shows the system overview.

The app “Screening” was for the child to perform under the administration of audiologist or trained personnel. The main function of the app is to perform the following test protocol (Preschool Audiometry Screening System, PASS).

After completing the test at the end of the screening test, there is a page reporting the scoring. Suggestions and recommendations, should the child fail and have to see a specialist, are also provided.

#### Speech Signal Measurement and Calibration

**Figure 2:**
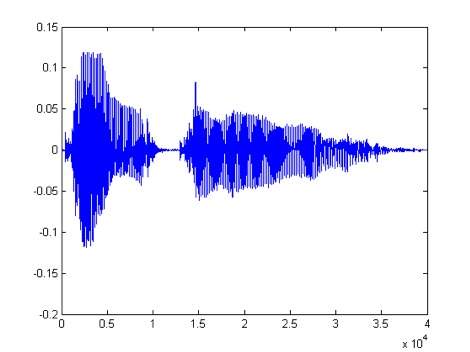
Speech signal.

Figure 2 presents an example of a speech signal. To calibrate the system to prepare for the trial, we first equalized the word files to have equal root mean square. We then calibrated each word individually at 20, 30, and 40 decibels hearing level (dBHL). This calibration was done by comparison to a reference standard speech audiometer.

**Figure 3:**
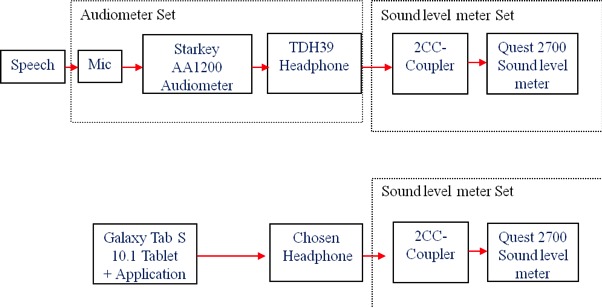
Calibration setups.

Figure 3 shows the setups. Through a 2-cc coupler, a sound level meter was used to measure the peak power (in A-weighted decibels, dBA) outputs from the standard audiometer and the tablet for each word at 20, 30, and 40 dBHL settings. The calibration coefficient calculated at each measuring point was basically an additional gain required that would make the tablet output the word with the same dBA peak when measured using the same sound level meter.

### Validity of the Software

#### Participants

We recruited 2 groups of 60 preschoolers from the northeastern part of Thailand. The first group was tested for the PASS speech audiometer version 1 using the commercial headphone, Creative EP-210. The second group was tested for the PASS speech audiometer version 2 using standard audiometric headphones, TDH39. The same inclusion and exclusion criteria were applied to both groups. The inclusion criteria were as follows: age between 4 and 5 years; use of standard Thai to communicate; and physical health, with the ability to cooperate with a medical examination. Candidates were excluded if they were uncooperative during conditioning play audiometry tests or they had incomplete conditioning play audiometry test results. An otolaryngologist examined the child’s ears without any intervention to clean the external ear. The PASS speech audiometry app tests were performed according to the test protocols in the quiet room.

#### Test Protocol in the Preschool Audiometry Screening System Speech Audiometry Version 1

**Figure 4:**
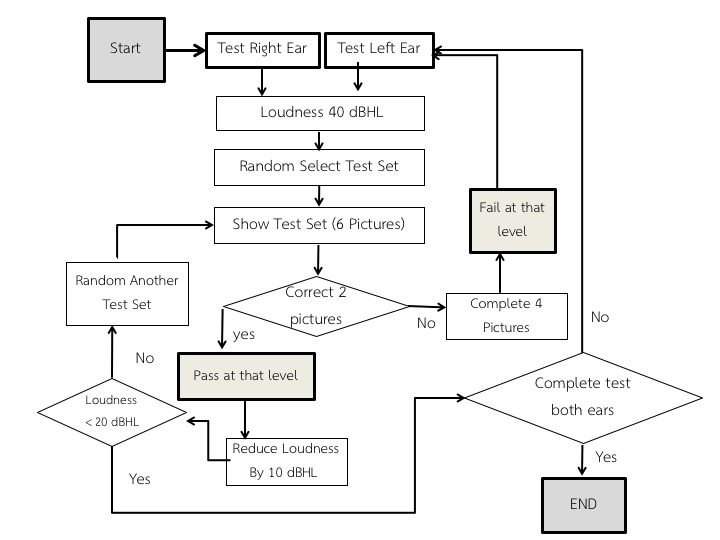
Screening protocol in Preschool Audiometry Screening System (PASS) speech audiometry app version 1. dBHL: decibels hearing level.

Starting from the right ear, the program randomly picks 1 set (6 pictures) out of an available 4 sets of pictures. The program first randomly outputs a 2-syllable word corresponding to 1 picture of the set at 40 dBHL. It then shows the 6 pictures to a user, who is required to pick the correct picture corresponding to the word from the picture set. The program continues with another word from the set. Only 4 words will be used for each speech level. Users move on to the next speech level if they answer correctly 2 of 3 or 4 times (passing the level) or 4 words out of the 6 have been used. In the latter case, the user is considered to have failed at that speech level. The test continues with the same procedure with lower power speech at 30 dBHL and, finally, at 20 dBHL. Other conditions that are observed are as follows: if the user fails the test at 40 dBHL or 30 dBHL, testing is stopped for the right ear and proceeds to the left ear and if the user passes all 3 levels, testing proceeds to the left ear. Figure 4 illustrates the entire testing protocol in the flowchart.

#### Test Protocol in The PASS Speech Audiometry Version 2

**Figure 5:**
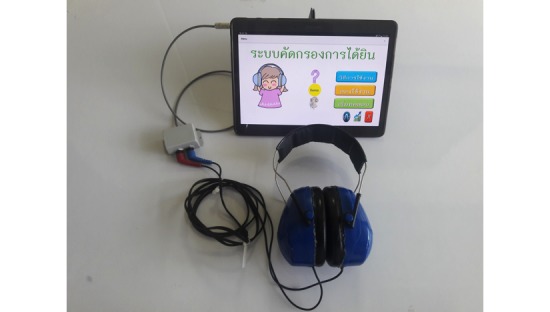
Tablet with TDH39 earmuffs.

For this phase of the trial, we tested the PASS speech audiometry app with standard audiometric headphones (TDH39). The headphones were embedded inside an earmuff with up to 20-dB ambient noise reduction, as it was intended for use in practical situations, such as school or normal rooms, rather than in an audiometric booth or sound-proof room, which are usually required for audiometric measurements. An additional Phono-to-Tip-Ring-Sleeve adaptor was required so that the TDH39 headphones could be plugged to the tablet. Figure 5 shows the complete system.

**Figure 6:**
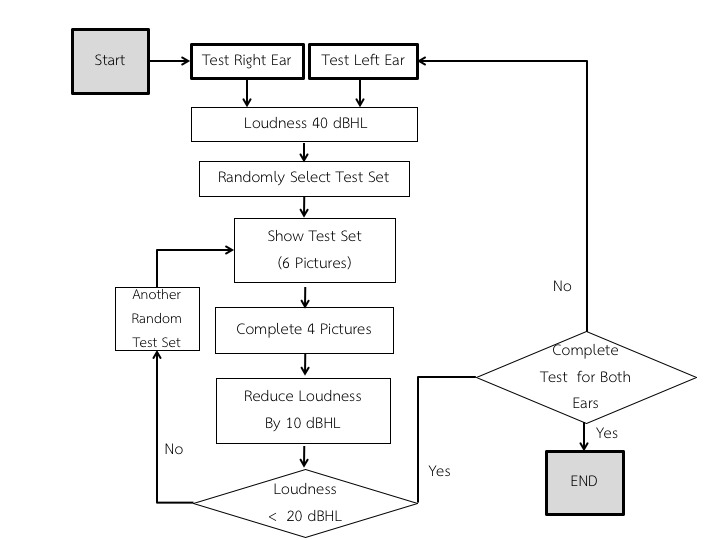
Screening protocol in Preschool Audiometry Screening System (PASS) speech audiometry app version 2. dBHL: decibels hearing level.

Starting from the right ear, the program randomly picks 1 set (6 pictures) out of the available 4 sets of pictures. The program first randomly outputs a 2-syllable word corresponding to 1 picture of the set at 40 dBHL. It then shows the 6 pictures to a child, who is required to select the correct picture corresponding to the word from the picture set. The program continues with another word from the set. The 40-dB sound is presented 4 times for each ear. The test continues with the same procedure with a softer speech at 30 dBHL and, finally, at 20 dBHL. Sounds were presented for each ear 12 times in total. Users will be considered as having passed the test at that speech level if they answer correctly, at least, 2 times out of 4. If a user cannot answer all 4 words correctly at each intensity, the user is considered to have failed at that level. The whole procedure is then repeated for the left ear. Figure 6 shows the protocol for PASS version 2.

#### Outcome Measurement

The audiologist who performed the test was not involved in the inventor team and was trained to use the PASS speech audiometry. Children were asked to hear the sound in each ear and point to the picture that they heard. The time used for the PASS speech audiometry was recorded.

The pediatric audiologist, who was blinded to the results of PASS speech audiometry, performed the conventional play audiometry. Children’s hearing threshold was evaluated with air-conduction pure-tone audiometry at 0.5, 1, 2, 3, and 4 kHz and with spondee words for the speech reception threshold (SRT) measurement in the standard sound-proof room. The procedure’s timing was recorded. Tympanometry was also carried out.

#### Statistical Analysis

The sensitivity and specificity of speech audiometry app to detect a mild hearing loss in either ear (pure-tone average, PTA, or SRT >20 dB) were tested. The mean difference between speech audiometry and conventional play audiometer for each protocol testing was then compared.

## Results

### Words Selected

**Figure 7:**
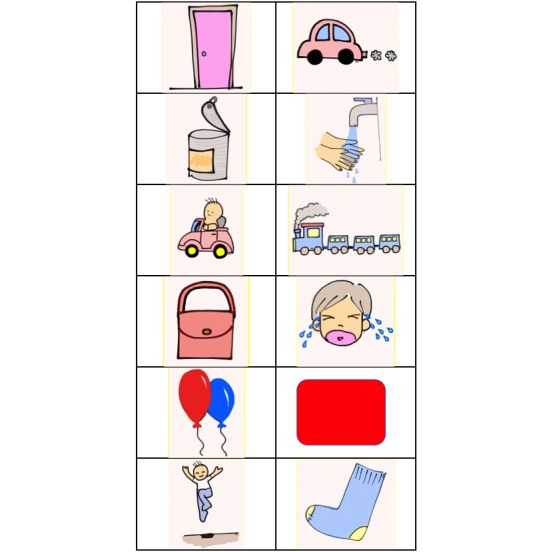
An example of picture sets.

For this part of the study, 63 children (51% (32/63) boys and 49% (31/63) girls) volunteered. Of these, 57% (36/63) were between 4 years, 7 months and 5 years of age, and 24% (15/63) were between 5 and 5.5 years. Each child would then go through the word selection trial as discussed in the previous section. Having all the results, audiologists finally grouped them into 4 sets of 6. Table 1 and Figure 7 present selected examples of the words and the corresponding pictures, respectively.

### PASS Speech Audiometry Version 1 Evaluation

There were 32 boys and 28 girls. The mean age was 4 years and 11 months. Although 2 children had impacted cerumen, 5 had otitis media; 10 children had a unilateral hearing loss, all of which was a mild hearing loss. No bilateral hearing loss was found. Commercial headphones, Creative EP-210 was used. The average time used for the speech audiometry program was 82.9 (SD 24.13) seconds (95% CI 76.67-89.13 seconds). The fastest time was 58 seconds, and the slowest was 195 seconds. The average time used for conventional play audiometry was 11.87 (SD 4.06) minutes (95% CI 10.79-12.87 minutes). The fastest was 6.3 minutes, and the slowest was 32.46 minutes. Table 2 shows the results in terms of the sensitivity and specificity compared with that of standard audiometric measurements.

### Results of PASS Speech Audiometry Version 2

**Figure 8:**
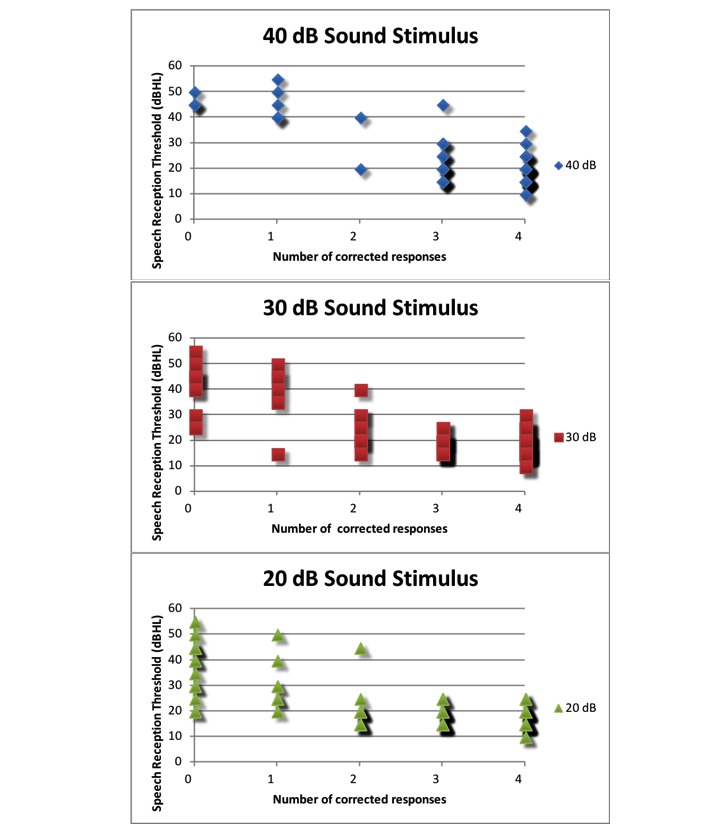
The relation of number of correction responses for each intensity and speech reception threshold. dBHL: decibels hearing level.

A total of 63 children participated in the second version trial; there were 22 boys and 38 girls, and the mean age was 4 years and 9 months. Four children had impacted cerumen, 15 had otitis media, and 14 had hearing loss. The unilateral hearing loss was found in 10 children, and bilateral hearing loss was found in 4 children, including 2 with the moderate hearing loss. The average time used for the speech audiometry program was 150.52 (SD 19.07) seconds (95% CI 145.71-155.32 seconds). The average time used for conventional play audiometry was 11.79 (SD 3.66) minutes (95% CI 10.85-12.71 minutes). Figure 8 shows the relation of the corrective score for each intensity and hearing threshold.

From Figure 8, we can see that a cutoff score of 2 would be appropriate to be used to classify pass or fail for each intensity test. Tables 3 and 4 show the sensitivity, specificity, and positive likelihood ratio of the PASS speech audiometry app under such a condition.

**Table 1:** Some of the test words.

Set 2	Set 3
door	car
can	wash hands
driving	train
bag	cry
balloon	red color
jump	sock

**Table 2:** The sensitivity and specificity of Preschool Audiometry Screening System speech audiometry app version 1 compared with that of Conventional Play Audiometry.

Test performance	Conventional play audiometry	
	Speech reception threshold 20 decibel, % (95% CI)	Pure-tone average_0.5-2 KHz_ 20 decibel, % (95% CI)
Sensitivity	62.50 (28.95-96.05)	60.00 (29.64-90.36)
Specificity	93.75 (89.27-98.23)	94.60 (90.30-98.79)
Positive likelihood ratio	10 (4.08-24.49)	11 (4.35-27.83)

**Table 3:** The sensitivity, specificity, and positive likelihood ratio of the Preschool Audiometry Screening System speech audiometry app version 2 compared with speech reception thresholds.

Stimuli and performance		SRT^a^>20 dB^b^, % (95% CI)	SRT>25 dB, % (95% CI)	SRT>30 dB, % (95% CI)	SRT>35 dB, % (95% CI)	SRT>40 dB, % (95% CI)
**PASS^c^-20**						
	Sensitivity	77.42 (60.19-88.60)	100 (83.18-100)	100 (78.47-100)	100 (77.19-100)	100 (72.25-100)
	Specificity	82.11 (73.20-88.52)	79.44 (70.83-86.01)	77.68 (69.12-84.40)	75.22 (66.52-82.26)	73.28 (64.57-80.49)
	LR+^d^	4.32 (2.70-6.93)	4.86 (3.35-7.06)	4.48 (3.17-6.33)	4.04 (2.93-5.57)	3.74 (2.77-5.06)
**PASS-30**						
	Sensitivity	65.63 (48.31-79.59)	94.74 (75.36-99.06)	100 (78.47-100)	100 (77.19-100)	100 (77.25-100)
	Specificity	93.62 (86.77-97.04)	91.59 (84.78-95.51)	91.07 (84.34-95.08)	87.61 (80.27-92.47)	86.21 (78.76-91.33)
	LR+	10.28 (4.56-23.20)	11.26 (5.97-21.24)	11.2(6.20-20.24)	8.07 (4.95-18.18)	7.25 (4.60-11.43)
**PASS-40**						
	Sensitivity	56 (37.07-73.33)	63.16 (41.04-80.85)	100 (78.47-100)	92.31 (66.69-98.63)	100 (67.56-100)
	Specificity	98.98 (94.44-99.82)	99.07 (94.90-99.83)	77.68 (69.12-84.40)	99.12 (95.16-99.84)	95.76 (90.46-98.18)
	LR+	54.88 7.52-397.74)	67.58 (9.32-489.84)	4.48 (3.17-6.33)	104.31 (14.73-738.72)	23.6 (10.01-55.65)

^a^SRT: speech reception threshold.

^b^dB: decibel.

^c^PASS: Preschool Audiometry Screening System.

^d^LR+: positive likelihood ratio.

**Table 4:** The sensitivity, specificity, and positive likelihood ratio of Preschool Audiometry Screening System speech audiometry app version 2 compared with average pure-tone air-conduction threshold of different frequencies.

Stimuli and performance		PTA^a^_0.5,1,2_ >20 dB^b^, % (95% CI)	PTA_0.5,1,2,4_ >20 dB, % (95% CI)	PTA_1,2,4_ >20 dB, % (95% CI)	PTA_0.5,1,2,4_ >25 dB, % (95% CI)	PTA_1,2,4_ >25 dB, % (95% CI)
**PASS^c^-20**						
	Sensitivity	55.00 (39.83-69.29)	68.99 (50.77-82.72)	76.67 (59.07-88.21)	90.0 (69.9-97.21)	89.47 (68.61-97.06)
	Specificity	96.51 (90.24-98.81)	97.93 (92.79-99.43)	95.83 (89.77-98.37)	93.40 (86.99-96.76)	92.52 (85.94-96.19)
	LR+^d^	15.77 (5.01-49.62)	33.45 (8.30-134.71)	18.4 (6.91-48.99)	13.63 (6.56-28.30)	11.97 (6.04-23.72)
**PASS-30**						
	Sensitivity	40.54.00 (26.35-56.51)	50.00 (33.15-66.85)	60.00 (40.74-76.60)	66.67 (45.37-82.81)	78.95 (56.67-91.49)
	Specificity	98.87 (93.91-99.8)	100.00 (96.15-100.0)	99.01 (94.60-99.83)	99.05 (94.80-99.83)	98.13 (93.44-99.49)
	LR+	36.08 (4.94-263.32)	N/A^e^	60.6 (8.39-473.30)	70 (9.72-503.92)	42.24 (10.50-169.98)
**PASS-40**						
	Sensitivity	29.72 (17.49-45.78)	40.00 (24.59-57.68)	36.67 (50.5-89.82)	57.14 (36.55-75.53)	63.16 (41.04-80.85)
	Specificity	100 (95.86-100)	100 (96.15-100)	100 (96.15-100)	100 (96.47-100.0)	100 (96.53-100)
	LR+	N/A	N/A	N/A	N/A	N/A

^a^PTA: pure-tone average.

^b^dB: decibel.

^c^PASS: Preschool Audiometry Screening System.

^d^LR+: positive likelihood ratio.

^e^N/A: not applicable.

**Figure 9:**
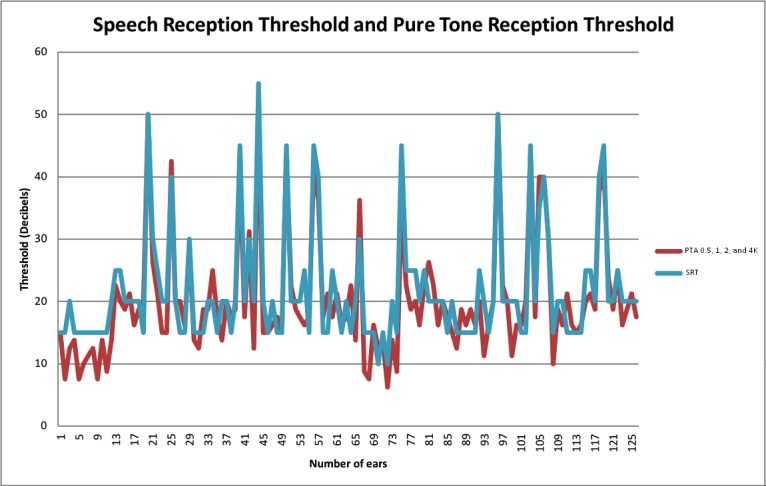
Speech reception threshold (SRT) and pure-tone average (PTA) threshold at 4 frequencies (0.5, 1, 2, and 4 kHz.) for each ear.

Under the cutoff score of 2 conditions, the sensitivity and specificity of the PASS speech audiometry app version 2 with the stimulus at a 20-dB sound intensity to detect the SRT >20 dBHL were 77.42% (95% CI 60.19-88.60) and 82.11% (95% CI 73.20-88.52), respectively. When using the SRT >25 dBHL, or PTA_0.5,1,2,4 kHz_>25 dBHL, or PTA_1,2,4 kHz_>25 dBHL as criteria for mild hearing loss, the sensitivity of the PASS speech audiometry app version 2 was greater and the specificity was similar. Increasing the sound stimulus intensity did not improve the sensitivity of the screening. Using the discrimination criterion of 20 dB, the sensitivity of the PASS speech audiometry version 2 was slightly variable among the frequencies of average pure-tone thresholds. If the PTA threshold at 500 Hz was omitted, the sensitivity was increased, and the specificity remained high. The sensitivity to screen mild hearing loss of PTA_1,2,4 kHz_>20 dBHL was 76.67% (95% CI 59.07-88.21), and the specificity was 95.83% (95% CI 89.77-98.37). Furthermore, the relation of the PTA threshold and SRT for individual ears was compared (Figure 9).

## Discussion

### Principal Findings

Hearing loss can be preventable and is much more manageable if detected early. To do so, we must equip our medical personnel, especially in low-resource countries, with tools that are appropriate, available, and accessible [19]. The tablet-based system, because of its relatively low cost and its internet connectivity, already fits the availability and accessibility demands. In Thailand, for example, the 3G cellular network has increasingly become the communication platform of choice for both voice and data, with penetration fast outstripping those offered by landlines. Potential reliability issues of the network can be addressed with appropriate system design. In PASS speech audiometry, data are stored locally and are only updated to the server when a connection is available.

The mobile hearing test apps, such as uHear [26] and hearScreen [27], use pure tones as the sound stimuli, whereas ShoeBOX Audiometry [28] uses warble tones [29]. The uHear was validated for the sensitivity in adults and the elderly, and it was found that it overestimated the PTA in all ears. The uHear was not suitable to screen mild hearing loss but had a potential benefit for the detection of moderate hearing loss [26,30-33]. The sensitivity and specificity to detect moderate hearing loss (PTA >40 dBHL) were 100% and 88%, respectively [30]. The hearScreen app was validated in 1070 school-age children to screen the hearing threshold of 25 dB at 1000 Hz [27,34]. The app had a sensitivity of 75% and a specificity of 98.5% for conventional screening and was 12.3% faster than that of conventional screening [34]. ShoeBOX Audiometry was validated in 80 children older than 4 years, and it was found that the obtained threshold in an uncontrolled environment did not correlate with the diagnostic threshold [29]. There was a sensitivity of 91.2% and a specificity of 57.8% when using a discrimination threshold of 30 dB. Using a discrimination threshold of 25 and 20 dB, the specificity decreased to 48.7% and 31%, respectively [29].

From the primary result of the PASS version 1 trial using SRT or PTA >20 dB as a criterion of mild hearing loss, the sensitivity of PASS speech audiometry was too low to be used as the hearing screening tool to detect mild hearing loss, though the specificity was very high. The test time was evidently very short, 8.6 times faster than that of conventional play audiometry, an encouraging result. We analyzed the results and considered 2 further changes so that we may learn more about its true potential. First, the test protocol could be modified to find the appropriate number of correct responses to determine pass or fail for each sound pressure level test; this may not be 2 of 4 as in the original protocol. Second, the headphone used in this trial should be the same standard used in conventional pure-tone audiometry for initial reference.

Using SRT or PTA_1,2,4_ >20 dB, the PASS speech audiometry app version 2 showed higher sensitivity and more specificity than version 1. While being able to complete the screening task much faster than when using standard tool, the PASS speech audiometry app version 2 had a sensitivity of 90% and 100%, and a specificity of 93.40% and 79.44% to differentiate children with normal hearing from those with the PTA or SRT >25 dB, respectively. Normally, the SRT is within 5 dB above PTA. Speech discrimination was increased if tested with the suprathreshold of sound stimuli. The PASS app uses the principle that the comprehension of sound will lead children to correctly choose the right picture. The sound stimulus may require higher intensity than that of the stimulus used for finding SRT.

For adults, the pure-tone, air-conduction threshold (PTA) at 500, 1000, and 2000 Hz >25 dBHL is considered to be a mild hearing loss. As mild hearing loss affects the academic performance in young school-age children, the criterion for mild hearing loss is set lower than that of adults [35]. The American Speech-Language-Hearing Association and the World Health Organization recommend using a PTA at 1000, 2000, and 4000 Hz >20 dBHL as screening levels for children. Dodd-Murphy et al investigated the use of 20 or 25 dBHL for screening educational significant hearing loss. The authors found that the sensitivity and specificity were different [36]; they suggested that pure-tone screening at 20 dBHL had the best combined sensitivity and specificity rates for educational significant hearing loss in children but unacceptable sensitivity when screening for PTA >25 [36].

The proposed system was based on the screening in Thai words for Thai children; however, we believe that the PASS app is universal and can be easily adopted for screening in other languages as well.

In the first trial, we used the same concept of the test protocol that we used in normal practice. If children could respond correctly to half of the test (at least 2 times), we could skip to the next loudness. In the second trial, we tested 4 times for each loudness regardless of the number of correct responses. We found that the cutoff point to determine pass or fail remained, at least, 2 correct responses. The mean test time used for each child in the second trial was twice as much as the time used for each in the first trial. For future improvement in terms of testing time and arrangement as that in the first test protocol may be considered.

Another point of consideration would be the selection of headphones. We were able to obtain good results using TDH39 headphones, which have a very flat response and loudness linearity that are important for quality speech measurement. This situation is not always the case for consumer headphones, and we should, therefore, be very specific if possible about which headphones should be used with the app. A pair of THD39 headphones can cost up to US $200, whereas reasonably priced headphones may be 5-10 times less expensive, making them more accessible.

**Figure 10:**
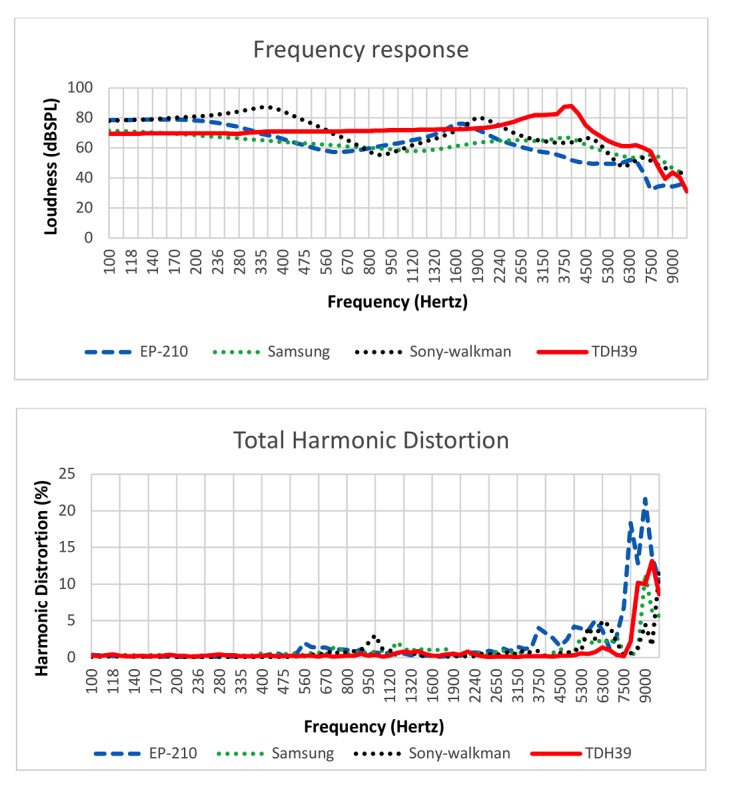
Electroacoustic measurements of sample headphones versus TDH39. dBSPL: decibels sound pressure level.

**Figure 11:**
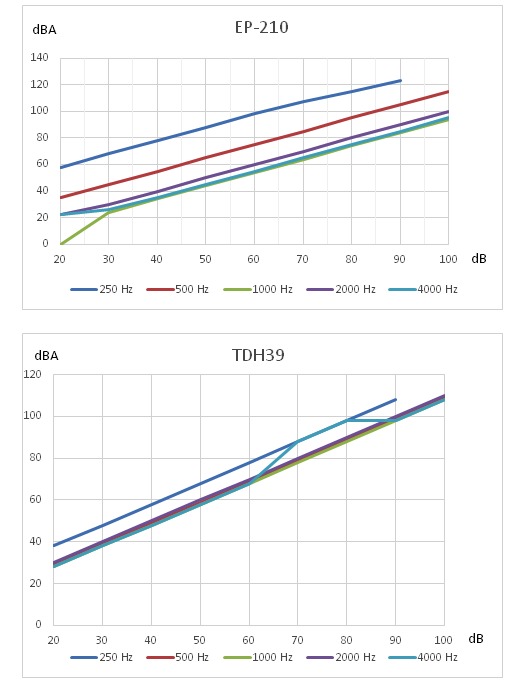
THD39 versus EP-210 loudness response. dBA: A-weighted decibels.

Figure 10 shows sample electroacoustic measurements of 3 midrange consumer headphones against those of TDH39. The stimulus input was a pure-tone sweep at 100 dBSPL, with each pair of headphones driving a 2-cc coupler used to simulate the load response of a simple ear canal [37]. High-intensity levels, such as 100 dBSPL, were used as an example here, as it would be the level that would approach the maximum level for many less expensive headphones such that they could begin to saturate. We can see that the low-end headphones had a fuzzier frequency response. At higher frequencies, the response of the midrange headphone was evidently nonideal. In the speech range (200-400 Hz), the frequency response was reasonably flat, making it a better candidate for the screening app. When comparing consumer headphones with TDH39, there remained nonlinearity in certain frequencies (Figure 11).

As the screening system must be accurate only for specific words at predefined loudness levels, we can simplify the calibration process by only finding the calibration coefficient at those points. This process could be done by adjusting the internal gain at each word-power level so that the output measurement by the sound level meter yields the same dBA as that measured when driving the TDH39 headphones. Masalski et al studied the reference sound level by means of biological calibration on 8620 devices representing models [38]. The reference sound levels were not very different among subjects and showed small deviations in the same model. Therefore, it is feasible to do the hearing test on mobile devices calibrated for the predefined reference sound level. Building up the library of supported headphones is the role of the app administration team, and we will continue to expand the usable headphone list. This situation is also true for the tablet itself, and we hope to be able to support Android tablets from various manufacturers.

### Limitations

A few limitations of this study deserve mention. First, because of the small sample size and the population that evidently had a low prevalence of hearing loss, some of the 95% CIs were rather wide. Future tests can be designed with increased sample sizes to cover more children with hearing loss. In addition, PASS version 2 was developed as an improvement to PASS version 1. For its trial evaluation, different populations with identical inclusion and exclusion criteria as those of PASS version 1 were used; this fact makes a direct comparison between the 2 trial results less straightforward. Finally, to practically reduce ambient noise to a minimum, an earmuff was used to cover the headphones. In cases where this is not possible, the true effect of using unprotected headphones on the screening performance must be studied.

### Future Directions

The screening program should add the function of headphone calibration before beginning the test to make it more feasible to accommodate other marketed devices. The test should be modified to make it more attractive to preschool toddlers. It would be ideal if the screening program could be complemented by other audiometry programs to offer more flexibility to the medical professional. Additional options could be standard pure-tone audiometry to study in detail the hearing loss characteristics of each. Conversely, it may be desirable to include a teleconsultant function that can bring a remote audiologist closer to the actual service field to validate hearing loss diagnostics, and any follow-up rehabilitation program can be done as close as possible to the primary practice. Being able to do these steps would address the low accessibility to qualified audiologists, which is a real concern in many countries. All this could be packaged together to form a teleaudiometry service that is an excellent example of mobile health. For the screening program itself, a new test algorithm that reduces the test time even further could be worth exploring. All these aspects are subjects of this work, which we will report in due course.

### Conclusions

This study demonstrates the potential use of a tablet-based system for rapid and mobile hearing screening. The system was shown to have good overall sensitivity and specificity. The idea can be easily adopted for systems based on other languages.

## Abbreviations

dBA: A-weighted decibels
dBHL: decibels hearing level
dBSPL: decibels sound pressure level
PASS: Preschool Audiometry Screening System
PTA: pure-tone average
SRT: speech reception threshold
